# Effect of Long-Term Taurine Supplementation on the Lipid and Glycaemic Profile in Adults with Overweight or Obesity: A Systematic Review and Meta-Analysis

**DOI:** 10.3390/nu17010055

**Published:** 2024-12-27

**Authors:** Qin Sun, Jieping Wang, Huanyu Wang, Hanhan Yu, Kang Wan, Fuyi Ma, Ru Wang

**Affiliations:** School of Exercise and Health, Shanghai University of Sport, Shanghai 200438, China; 2111516009@sus.edu.cn (Q.S.); wangjieping94@126.com (J.W.); 2211516021@sus.edu.cn (H.W.); 2421516013@sus.edu.cn (H.Y.); kk999ww@163.com (K.W.); 18505093326@163.com (F.M.)

**Keywords:** taurine supplementation, obesity, glycemic control, insulin sensitivity

## Abstract

Background: Taurine has been demonstrated to regulate and improve metabolic health. However, physiological and pathological differences among individuals with overweight or obesity may result in varied responses to taurine supplementation. This study aims to estimate the effects of long-term taurine supplementation on blood lipids, glycemia, and insulin sensitivity in adults with overweight or obesity through a systematic review and meta-analysis. Methods: The literature search was based on six databases (Web of Science, PubMed, Scopus, EMBASE, Cochrane, and SPORTDiscus) up to October 2024. Subgroup analyses were performed based on daily taurine intake dosage (<3 g or 3 g), overweight (BMI 25–29.9 kg/m^2^), and obesity (BMI ≥30 kg/m^2^). Results: The final number of studies that met the inclusion criteria was 9 RCTs. The overall analysis showed that taurine supplementation significantly decreased TG (WMD = −0.56 mg/dL, 95% CI: −0.92 to −0.2, *p* = 0.002, *I*^2^ = 63%), TC (WMD = −0.71 mg/dL, 95% CI: −1.17 to −0.25, *p* = 0.002, *I*^2^ = 73%), and fasting insulin (WMD = −2.15 µU/mL, 95% CI: −3.24 to −1.06, *p* = 0.0001, *I*^2^ = 9%). In the subgroup analysis, long-term taurine intake led to BMI improvement in overweight adults (WMD = −1.14 kg/m^2^, 95% CI: −1.81 to −0.47, *p* = 0.0008, *I*^2^ = 0%). Meanwhile, improvements in HbA1c (WMD = −0.33%, 95% CI: −0.53 to −0.12, *p* = 0.002, *I*^2^ = 16%) and HOMA-IR (WMD = −0.91, 95% CI: −1.74 to −0.08, *p* = 0.003, *I*^2^ = 54%) were observed only in obese participants following taurine supplementation. Additionally, the long-term intake of 3 g of taurine significantly improved HbA1c (WMD = −0.37%, 95% CI: −0.61 to −0.13, *p* = 0.003, *I*^2^ = 0%) and FPG levels (WMD = −7.14 mg/dL, 95% CI: −12.53 to −1.74, *p* = 0.003, *I*^2^ = 70%) in overweight/obesity. Conclusions: Long-term taurine supplementation is particularly effective in improving glycemic control and insulin sensitivity in obesity. Furthermore, higher doses of taurine (3 g per day) demonstrate even greater improvements in glycemic control.

## 1. Introduction

Obesity, as defined by the World Health Organization (WHO), is a pathophysiological condition characterized by abnormal or excessive fat accumulation that causes significant health risks [1,2]. Overweight and obesity are typically classified using body mass index (BMI) [3]. According to the WHO, overweight is defined as a BMI between 25 and 29.9 kg/m^2^, while obesity is defined as a BMI of 30 kg/m^2^ or higher [4]. Epidemiological studies indicate a continuous global rise in the incidence of obesity, with ~2 billion overweight/obese individuals worldwide, representing ~30% of the global population [1]. In fact, Obesity serves as a major risk factor for metabolic disorders, including atherosclerosis, metabolic syndrome, and type 2 diabetes mellitus (T2DM) [5]. Furthermore, obesity is associated with metabolic dysfunctions such as hyperglycemia, dyslipidemia, altered lipid profiles, and reduced insulin sensitivity [6,7]. Systemic lipid deposition and central obesity (referring to fat tissue deposited around the trunk, including visceral fat surrounding central organs) further impair cardiovascular structure and function, thereby increasing the risk of all-cause mortality [8]. As a multifaceted and growing global health challenge, obesity and its related complications impose a substantial burden on healthcare systems worldwide.

Taurine (2-aminoethanesulfonic acid), a sulfur-containing semi-essential amino acid, is widely distributed in various tissues and organs, particularly abundant in major metabolic organs (such as the heart and skeletal muscle) [9,10]. It can be synthesized endogenously via pathways involving cysteine and methionine, or obtained from dietary sources such as seafood, meat, and energy drinks [11,12]. Taurine is known for its beneficial roles in multiple metabolic and physiological processes, including energy metabolism, antioxidation, and anti-inflammation [13,14]. Given these properties, taurine has been widely used as a performance-enhancing agent in sports. For example, taurine supplementation has been shown to enhance both aerobic and anaerobic performance, including delayed onset of fatigue, increased strength, and enhanced explosive power [15,16,17]. Moreover, taurine reduces muscle damage by decreasing inflammatory markers and alleviates exercise-induced fatigue by lowering lactate and creatine kinase levels [15,16,17].

Recently, there has been increasing attention paid to the role of taurine in regulating and boosting metabolic homeostasis. Two previous studies have demonstrated that taurine supplementation can significantly reduce important metabolic indicators such as blood lipids, blood pressure and FBG (fasting blood glucose) in people with metabolic diseases [18,19,20,21]. Notably, Wei W and other researchers have recently revealed in animal experiments that N-acetyltaurine (the secondary metabolite of taurine) can regulate body weight and control energy balance, demonstrating the potentially important clinical value of taurine in the resistance to obesity [22]. Although preclinical and clinical studies have shown the benefits of taurine on ameliorating metabolic abnormalities, the results of studies on taurine supplementation for improving overweight/obesity are inconsistent. For example, a meta-analysis on the effect of taurine supplementation on blood pressure and blood lipid status in people with obesity found that taurine may not improve BMI and FPG in obese individuals [23]. The uncertainty in these results can be attributed to various factors, including small sample sizes, differences in taurine intake doses, and variations in disease states.

Therefore, we conducted this systematic review and meta-analysis to comprehensively estimate the potential effects of taurine in overweight/obesity management. The objectives of this study were to examine the impact of long-term taurine supplementation on blood lipid levels, glycemia, and insulin sensitivity in overweight/obese adults.

## 2. Methods

### 2.1. Overview

The meta-analysis was conducted in accordance with the Preferred Reporting Items for PRISMA guidelines and the Cochrane Collaboration Handbook [24]. A literature search was performed to identify relevant studies published up to October 2024 across six databases: Web of Science, PubMed, Scopus, EMBASE, Cochrane, and SPORTDiscus. Furthermore, this study has been registered with the International Prospective Register for Systematic Reviews (PROSPERO; registration number: CRD42024598354). An amalgamation of MeSH and non-MeSH terms were employed: (‘Taurine’ OR ‘Taufon’ OR ‘Tauphon’ OR ‘Taurine, Monopotassium Salt’) AND (‘Obesity’ OR ‘Overweight’). Additional eligible studies were identified through supplementary approaches, including hand-searching and reviewing reference lists.

### 2.2. Inclusion and Exclusion Criteria

The selected studies were included according to Population, Intervention, Comparison, Outcomes, and Study model (Table 1). Intervention studies conducting taurine ingestion were included. Studies were excluded if: (a) taurine is not the only complementary supplement; (b) placebo data were not available; (c) the full-text data and detailed results were insufficient.

Subgroup analysis was performed to distinguish the effects of overweight/obesity on blood lipid, glycemia, and insulin sensitivity after taurine supplementation. Blood lipids were measured by TG (triglycerides, mg/dL), TC (total cholesterol, mg/dL), LDL-C (low-density lipoprotein, mg/dL), and HDL-C (high-density lipoprotein, mg/dL). Glycemia was determined as HbA1c (glycated hemoglobin, %) and FPG (mg/dL). Insulin sensitivity was evaluated by HOMA-IR (homeostatic model assessment for insulin resistance) and fasting insulin (µU/mL).

**Table 1:** Study inclusion criteria based on PICOS model.

Parameter	Inclusion Criteria
Population	Adults (≥18 years old) with overweight (body mass index [BMI] ≥ 25–29.9 kg/m^2^) or obesity (BMI ≥ 30 kg/m^2^) [4]
Intervention	Long-term (≥2 weeks) taurine intervention
Comparator	Placebo
Outcome	Indicators of overweight/obesity or insulin sensitivity
Study design	Randomized control trials

Abbreviation: PICOS = Population, Intervention, Comparator, Outcome, Study design.

### 2.3. Data Extraction

The preliminary review of the records from all databases and the eligibility of the studies was conducted by the 2 separate investigators (KW and FYM), and the disagreements resolved by a third investigator (HYW). Then, results were confirmed by the 2 separate investigators (QS and JPW), and any disagreements about study inclusion or exclusion are resolved through discussion with the other investigator (RW). The third investigator reviewed the relevant study details and provided a final decision, which was accepted by all parties. After removing duplicates, all remaining articles were filtered by title and abstract (stage 1) and then by full-text specific content (stage 2). Records were imported into Endnote (Version X9.3.3; Clarivate Analytic, Philadelphia, PA, USA) and performed automated and manual screening. After finalizing the screening of included studies, the data were categorized based on participant characteristics (sample size, age, and BMI) and intervention type. All study outcome data were expressed using the weighted mean difference (WMD).

### 2.4. Quality and Risk of Bias Assessments

The quality of the studies included in the analysis was assessed using the updated Cochrane Risk of Bias tool for randomized trials, which evaluates five primary aspects: (1) randomization process, (2) deviations from intended interventions, (3) missing outcome data, (4) measurement of the outcome, and (5) selection of the reported result. The methodological quality and risk of bias of each eligible trial were independently evaluated by two investigators, Qin Sun and Jieping Wang, who were responsible for the assessment. Disagreements were resolved through consultation with a third investigator, Ru Wang, who helped reconcile the differences [25].

### 2.5. Statistical Analysis

The meta-analysis was conducted using Review Manager software (RevMan Version 5.4.1; Cochrane, London, UK). Given the use of continuous data (mean ± SD), weighted mean differences (WMD) with 95% confidence intervals (95% CI) were used for the final analysis. Means and SD were extracted for both the placebo and taurine groups. Subgroup analyses were performed for outcomes in relation to the overweight/obesity and different doses of taurine. Forest plots were produced to show WMD, SD, and the overall effect of Z score. All studies included in the analysis used the same scale for the outcomes. In cases where the original units differed, we performed appropriate unit conversions to ensure uniformity and consistency in the meta-analysis. In the case of unit inconsistency, unit conversion is performed to ensure consistency of results between different studies.

To assess heterogeneity, we used tau-squared (τ^2^), *χ*^2^ Cochran’s Q (*x*^2^) tests as well as *I*^2^ statistics. A value of τ^2^ > 1 indicates inter-study variability. The *Q* test was used to measure variation around the weighted mean, in which *p* < 0.10 was considered significant heterogeneity [26]. The *I*^2^ statistic was used to assess the consistency of effects across studies, with the interpretation of *I*^2^ as follows: (a) *I*^2^: 0–29%, no important heterogeneity, (b) *I*^2^: 30–49%, moderate heterogeneity, (c) *I*^2^: 50–74%, substantial heterogeneity, and (d) *I*^2^: 75–100%, considerable heterogeneity. When the heterogeneity was not significant (*I*^2^ ≤ 50% and *p* ≥ 0.1), the fixed effect model was used for meta-analysis. However, when heterogeneity was significant (*I*^2^ > 50% or *p* < 0.1), the random-effects model was used. *p* < 0.05 was considered statistically significant [27].

## 3. Results

### 3.1. Literature Search

**Figure 1:**
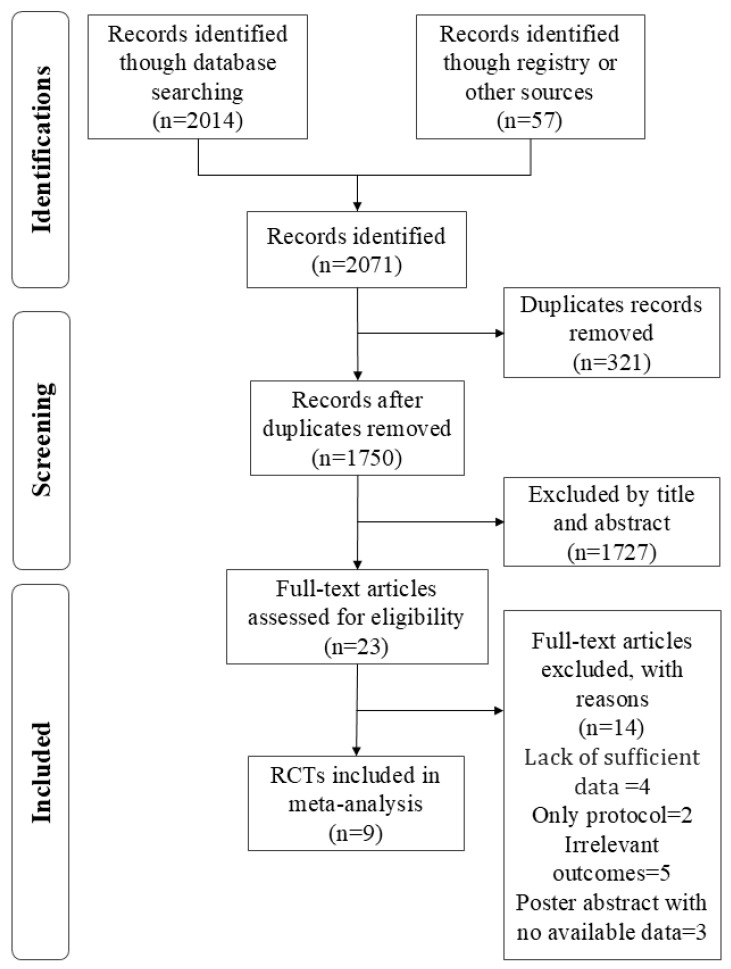
The Preferred Reporting Items for Systematic Reviews and Meta-analyses (PRISMA) flow diagram defined the electronic search and selection process.

Nine eligible articles were included in the current quantitative analysis after screening. A total of 2071 intervention studies were retrieved from six databases (Web of Science, PubMed, Scopus, EMBASE, Cochrane, and SPORTDiscus) shown in Figure 1. After a screening phase (including duplicate articles, titles, and abstracts), 2048 duplicate or unrelated articles were excluded. Fourteen articles were excluded from this meta-analysis for the following reasons: (a) insufficient data [28,29,30,31], (b) only protocol [32,33], (c) irrelevant outcomes [34,35,36,37,38], (d) poster abstract with no available data [39,40].

### 3.2. Study Characteristics

The characteristics of the studies included in the meta-analysis are summarized in Table 2. A total of nine RCTs (randomized controlled trials) were included, which were published between 2004 and 2022 [18,19,20,21,41,42,43,44,45]. All the study samples comprised adults (aged 20.3 ± 1.7 to 53.08 ± 8.8) with the BMI ≥ 25 kg/m^2^. Among them, participants in four studies were obese with T2DM [18,19,21,42], while one study included obese participants with type 1 diabetes [44]. The dosages of taurine supplementation varied from 1 g to 3 g, and was administered continuously for periods varying from 2 weeks to 12 weeks. In the current meta-analysis, indicators related to overweight/obesity and insulin sensitivity include BMI, TG, TC, LDL-C, HDL-C, fasting insulin, HbA1c, and FPG.

### 3.3. Quality Assessment in Individual Studies

Among the included studies, none was identified as having a high risk of bias. There were two articles with moderate-risk bias [20,41] and seven articles with low-risk bias [18,19,21,42,43,44,45]. The results of the study quality assessment are shown in Supplementary Figures S7 and S8.

**Table 2:** Characteristics of studies included in the meta-analysis.

Study	Information of Participants			Intervention (Dosage)	Outcome	Conclusion
	Sample Size and Sex (Male/Female)	Age (Year) (Mean ± SD)	BMI (kg/m^2^)			
Esmaeili F et al. (2021) [21]	46 participants with T2DM (14/32)	43.1 ± 7.1	31.10 ± 3.0	3 g/day taurine, 8 weeks	FPG, HbA1c, Fasting insulin, HOMA-IR, TG, TC, LDL-C, HDL-C	Glycemic control and advanced glycation end products were improved by taurine.
Maleki V et al. (2020) [19]	45 participants with T2DM (13/32)	42.7 ± 6.9	30.7 ± 3.4	3 g/day taurine, 8 weeks	Weight, BMI, FPG, HbA1c, Fasting insulin, HOMA-IR, TG, TC, LDL-C, HDL-C	Taurine improved glycemic indexes and lipid profiles in individuals with T2DM.
Zhang M et al. (2004) [43]	30 participants (14/16)	20.3 ± 1.6	26.6 ± 3.5	3 g/day taurine, 7 weeks	Weight, BMI, FPG, TG, TC, HDL-C	Taurine improves lipid metabolism.
Brons C et al. (2004) [42]	20 participants (no mention)	40.0 ± 8.0	27.9 ± 2.7	3 g/day taurine, 8 weeks	Weight, BMI, FPG, HOMA-IR, TG, TC, HDL-C, LDL-C, HbA1c	Taurine did not improve insulin secretion, insulin sensitivity, or blood lipid levels.
Rosa FT et al. (2014) [45]	16 female participants (0/16)	32.0 ± 8.0	47.0 ± 6.0	3 g/day taurine, 8 weeks	Weight, BMI, FPG, Fasting insulin, HOMA-IR	Taurine improved metabolic health in obesity women
Moloney MA et al. (2010) [44]	19 male participants with T1DM (19/0)	28.0 ± 2.0	27.9 ± 1.2	1.5 g/day taurine, 2 weeks	FPG, HbA1c, TG, TC, HDL-C, LDL-C	Taurine assisted in reversing early detectable vascular abnormalities in young male diabetics.
Haidari F et al. (2020) [20]	38 female participants (0/38)	36.1± 8.6	32.9 ± 2.7	3 g/day taurine, 8 weeks	Weight, BMI, FPG, Fasting insulin, HOMA-IR, TG, TC, LDL-C, HDL-C	Taurine with a weight-loss diet can improve the lipid profile and metabolic risk factors.
Falah Hassan S et al. (2019) [41]	80 participants with T2DM (46/34)	49.5 ± 3.5	33.0 ± 5.5	1 g/day taurine, 12 weeks	Weight, BMI, FPG, HbA1c	Taurine did not improve glycemic control or HbA1c levels.
Moludi J et al. (2022) [18]	120 participants with T2DM (97/23)	52.6 ± 8.5	28.0 ± 7.0	3 g/day taurine, 8 weeks	Weight, BMI, FPG, Fasting insulin, HOMA-IR, TG, TC, LDL-C, HDL-C, HbA1c	Taurine reduced insulin levels, HOMA-IR, oxidative stress, inflammation, and endothelial markers in individuals with T2DM.

Abbreviation: T2DM = type 2 diabetes mellitus; T1DM = type 1 diabetes mellitus; BMI = body mass index; FPG = fasting plasma glucose; HOMA-IR = homeostatic model assessment for insulin resistance; TG = triglyceride; TC = total cholesterol; LDL-C = low density lipoprotein cholesterol; HDL-C = high density lipoprotein cholesterol; HbA1c = glycated hemoglobin.

### 3.4. The Effect of Taurine on the Body Composition

**Figure 2:**
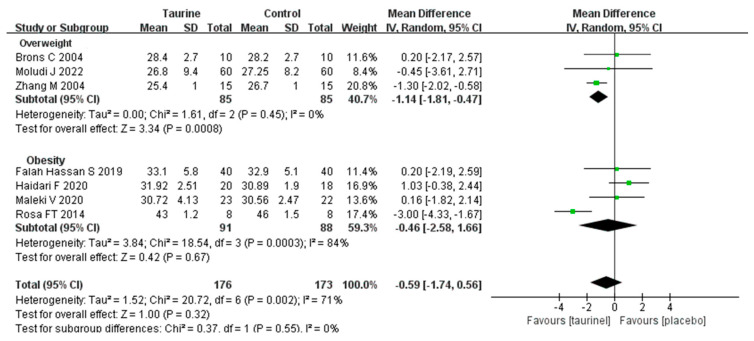
Forest plot of standardized mean difference for taurine intake on BMI. 95%CI = 95% confidence interval; *df* = degree of freedom; *I*^2^ = inconsistency between studies; BMI = body mass index [18,19,20,41,42,43,45].

A total of seven studies measured differences in BMI in overweight/ obese adults after taurine supplementation (Figure 2) [18,19,20,41,42,43,45]. Taurine supplementation did not have a significant effect on the improvement in BMI (WMD = −0.59 kg/m^2^, 95%CI: −1.74 to 0.56, *p* = 0.32, *I*^2^ = 71%). However, subgroup analyses revealed that taurine consumption significantly improved BMI in overweight adults (WMD = −1.14 kg/m^2^, 95%CI: −1.81 to −0.47, *p* = 0.0008, *I*^2^ = 0%) while having no discernible positive effect on BMI in obesity (WMD = −0.46 kg/m^2^, 95%CI: −2.58 to 1.66, *p* = 0.67, *I*^2^ = 84%).

Furthermore, six studies were subjected to a subgroup analysis of weight (Figure S1) [18,19,20,41,42,43,45]. Results showed that taurine supplementation had a positive effect on body weight in overweight adults (WMD = −2.67 kg, 95%CI: −5.35 to 0.00, *p* = 0.05, *I*^2^ = 37%) compared with obese individuals (WMD = −1.22 kg, 95%CI: −4.66 to 2.23, *p* = 0.49, *I*^2^ = 0%).

### 3.5. The Effect of Taurine on the Blood Lipid

**Figure 3:**
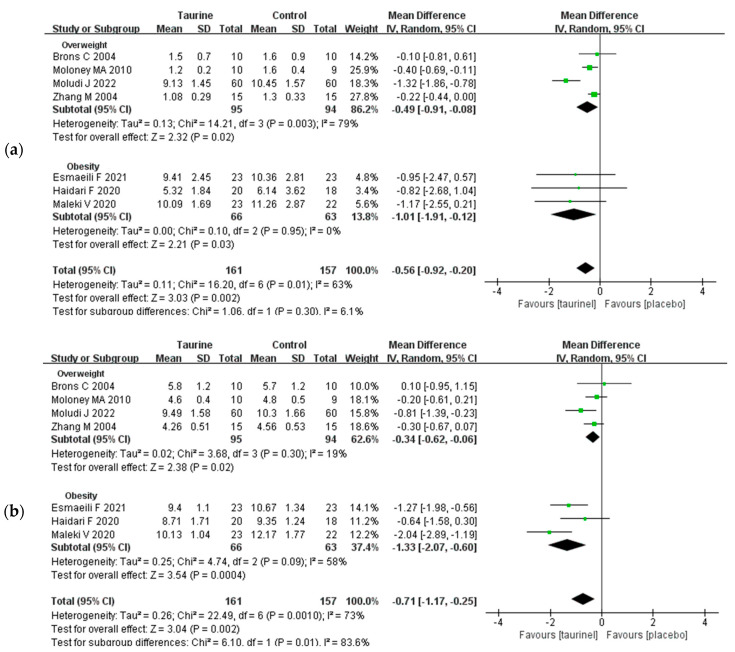
(**a**) Forest plot of standardized mean difference for taurine intake on TG; (**b**) forest plot of standardized mean difference for taurine intake on TC. 95%CI = 95% confidence interval; *df* = degree of freedom; *I*^2^ = inconsistency between studies; TG = triglyceride; TC = total cholesterol [18,19,20,21,42,43,44].

Seven studies measured the impact of taurine supplementation on TG levels in overweight/ obese adults (Figure 3a) [18,42,43,44]. Despite the variations in the subjects’ levels of overweight and obesity, taurine supplementation significantly improved TG (WMD = −0.56 mg/dL, 95% CI: −0.92 to −0.2, *p* = 0.002, *I*^2^ = 63%). A subgroup analysis showed that taurine consumption significantly improved TG in overweight adults (WMD = −0.49 mg/dL, 95% CI: −0.91 to −0.08, *p* = 0.02, *I*^2^ = 79%), and improved TG more significantly in those who were obese (WMD = −1.01 mg/dL, 95% CI: −1.91 to −0.12, *p* = 0.03, *I*^2^ = 0%). Similarly, seven studies examined variations in TC in persons who were overweight or obese following taurine supplementation (Figure 3b) [18,42,43,44]. Overall, TC was significantly improved by taurine supplementation (WMD = −0.71 mg/dL, 95% CI: −1.17 to −0.25, *p* = 0.002, *I*^2^ = 73%). There was a considerable drop in TC when the analysis was limited to studies involving either overweight (WMD = −0.34 mg/dL, 95% CI: −0.62 to −0.06, *p* = 0.02, *I*^2^ = 19%) or only obese individuals (WMD = −1.33 mg/dL, 95% CI: −2.07 to −0.60, *p* = 0.0004, *I*^2^ = 58%).

In addition, a total of six studies evaluated the effect of taurine supplementation on LDL-C in overweight/obese subjects (Figure S2) [18,42,44]. A subgroup analysis revealed that, whereas taurine supplementation significantly improved LDL-C levels in obese individuals (WMD = −0.98 mg/dL, 95% CI: −1.74 to −0.22, *p* = 0.01, *I*^2^ = 62%), it had no significant effect on overweight individuals (WMD = −0.44 mg/dL, 95% CI: −0.98 to −0.10, *p* = 0.11, *I*^2^ = 62%). Conversely, seven investigations evaluated the impact of taurine supplementation on HDL-C in individuals who were overweight or obese (Figure S3) [18,19,20,21,42,43,44]. A subgroup analysis found that taurine supplementation significantly increased HDL-C levels in overweight participants (WMD = 0.15 mg/dL, 95% CI: 0.09 to 0.21, *p* < 0.00001, *I*^2^ = 6%) but had no significant impact on HDL-C levels in obese participants (WMD = 0.02 mg/dL, 95% CI: −0.09 to 0.13, *p* = 0.08, *I*^2^ = 60%).

### 3.6. The Effect of Taurine on the Glycemic Control

**Figure 4:**
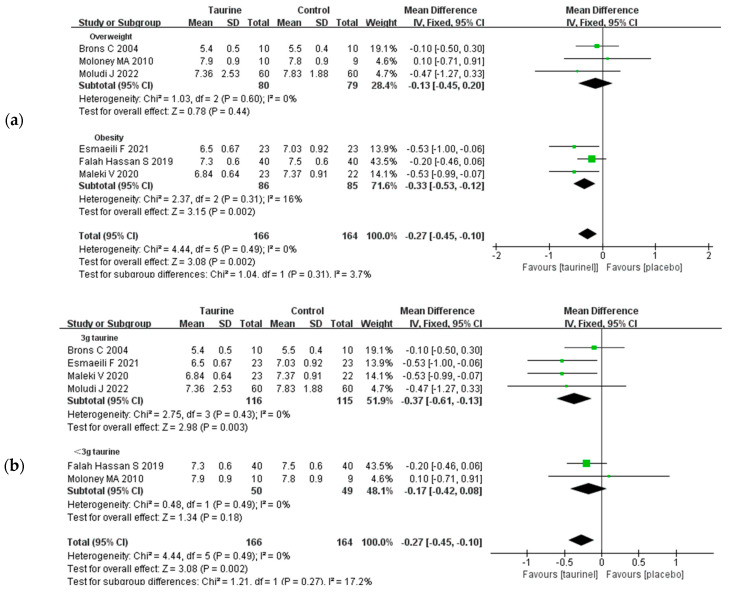
(**a**) Forest plot of standardized mean difference for taurine intake on HbA1c; (**b**) forest plot of standardized mean difference for taurine dose on HbA1c. 95%CI = 95% confidence interval; *df* = degree of freedom; *I*^2^ = inconsistency between studies; HbA1c = glycated haemoglobin [18,19,21,41,42,44].

The impact of taurine supplementation on HbA1c in overweight or obese individuals was examined in six studies (Figure 4a) [19,20,21,41,42,44]. Taurine intake did not significantly reduce HbA1c in overweight people (WMD = −0.13%, 95% CI: −0.45 to 0.20, *p* = 0.60, *I*^2^ = 0%), but it did demonstrate a significant reduction in HbA1c in obese adults (subgroup analysis: WMD = −0.33%, 95% CI: −0.53 to −0.12, *p* = 0.002, *I*^2^ = 16%). A subgroup analysis revealed that taurine intake up to 3 g/day (WMD = −0.37%, 95% CI: −0.61 to −0.13, *p* = 0.003, *I*^2^ = 0%) could significantly downregulate HbA1c, while an intake below 3 g/day (WMD = −0.17%, 95% CI: −0.42 to 0.08, *p* = 0.49, *I*^2^ = 0%) had no significant effect on HbA1c (Figure 4b) [18,19,21,41,42,44].

In addition, nine studies examined the effects of taurine supplementation on FPG in individuals who were overweight or obese (Figure S4) [18,19,20,21,41,42,43,44,45]. In general, taurine improves FPG in overweight/obese individuals (WMD = −5.60 mg/dL, 95% CI: −10.09 to −1.12, *p* = 0.003, *I*^2^ = 66%). In contrast to overweight persons (WMD = −4.16 mg/dL, 95% CI: −10.31 to 1.99, *p* = 0.20, *I*^2^ = 35%), a subgroup analysis revealed that taurine supplementation further improved FPG levels in individuals with obesity (WMD = −7.16 mg/dL, 95% CI: −14.45 to −0.14, *p* = 0.05, *I*^2^ = 78%). Additionally, a subgroup analysis was carried out in nine trials based on the amount of taurine consumed daily (Figure S5) [18,19,20,21,41,42,43,44,45]. The findings demonstrated that, whereas a daily consumption of less than 3 g of taurine had no significant influence on FPG (WMD = 5.84 mg/dL, 95% CI: −22.29 to 33.35, *p* = 0.22, *I*^2^ = 35%), a daily consumption of 3 g of taurine could considerably lower FPG (WMD = −7.14 mg/dL, 95% CI: −12.53 to −1.74, *p* = 0.003, *I*^2^ = 70%).

### 3.7. The Effect of Taurine on the Insulin Resistance

**Figure 5:**
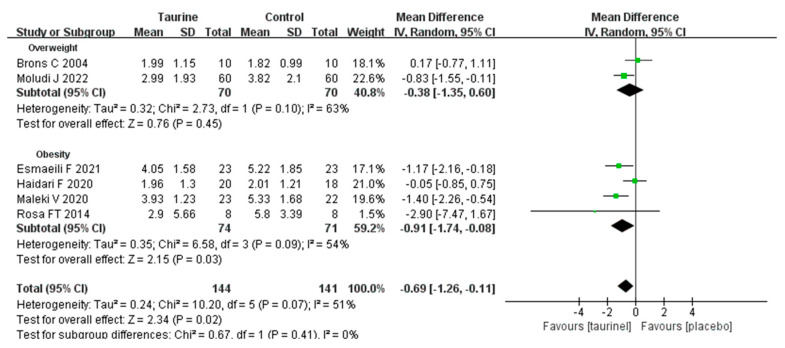
Forest plot of standardized mean difference for taurine intake on HOMA-IR; 95%CI = 95% confidence interval; *df* = degree of freedom; *I*^2^ = inconsistency between studies; HOMA-IR = homeostatic model assessment for insulin resistance [18,19,20,21,42,45].

A total of six studies measured the effect of taurine supplementation on HOMA-IR in overweight/obese adults (Figure 5) [18,19,20,21,42,45]. The results demonstrated that the HOMA-IR baseline was higher in obese adults (mean: 4.59 ± 2.19) than in overweight adults (mean: 2.82 ± 1.64).

A subgroup analysis revealed a significant beneficial effect of taurine supplementation on HOMA-IR in obese subjects (WMD = −0.91, 95% CI: −1.74 to −0.08, *p* = 0.003, *I*^2^ = 54%). On the contrary, the intake of taurine for overweight adults HOMA-IR had no significant influence (WMD = −0.38, 95% CI: −1.35 to 0.60, *p* = 0.45, *I*^2^ = 63%). In addition, five studies also showed that taurine supplementation made a significant improvement to fasting insulin in overweight/obese individuals (WMD = −2.15 95% CI: −3.24 to −1.06, *p* = 0.0001, *I*^2^ = 9%) (Figure S6) [21].

## 4. Discussion

In this comprehensive quantitative review, we examined the effects of long-term taurine supplementation on metabolic markers (including lipids, glucose, and insulin sensitivity) in overweight and obese individuals. In the present meta-analysis, we found that long-term taurine supplementation significantly reduced TG and TC levels in overweight and obese individuals. Nevertheless, a significant decrease in BMI was observed only in people with overweight. Furthermore, we found that long-term taurine supplementation, especially at a higher dose (3 g/day), could significantly decrease HbA1c and FPG levels over time in obesity but not in those who were overweight. In addition, taurine supplementation further increased insulin sensitivity (HOMA-IR was significantly reduced) in obese participants. These findings suggest that taurine supplementation may differentially enhance metabolic health depending on the degree of obesity.

The present meta-analysis reveals that taurine intake is effective in reducing body weight and BMI specifically in overweight adults. This effect is attributed to taurine’s role in regulating lipid metabolism and promoting energy expenditure. Previous studies have shown that taurine reduces fatty acid biosynthesis by directly or indirectly reducing lipid synthase activity in adipose tissue or liver [18,19,20,45]. In addition, taurine stimulates the synthesis of bile acids, thereby promoting cholesterol catabolism [46]. Meanwhile, taurine supplementation can upregulate the expression of metabolic genes, including peroxisome proliferator-activated receptor α(PPARα), PPARγ and peroxisome proliferator-activated receptor gamma coactivator-1α(PGC-1α), and promote energy expenditure [47]. Consistent with these mechanisms, our findings indicate that sustained taurine supplementation also improved lipid metabolism by significantly lowering both blood TG and TC levels. However, the effect of taurine on weight loss or BMI reduction was not significant in obese individuals. This difference in results may be related to the altered body composition and complex metabolic milieu in obese people. The distribution and concentration of medications in the body, which in turn govern and may alter the actual drug effects, can be greatly impacted by changes in biological anatomy [48]. Previous studies have shown that obesity-induced changes in body composition can impair the effective concentration of supplements, affecting their efficacy [48]. As a hydrophilic drug, the distribution of taurine in the body is primarily influenced by the water content of body fluids and tissues [49]. With increasing obesity, the body’s fat percentage rises while the water content decreases correspondingly, potentially reducing taurine’s effective concentration in major metabolic organs such as adipose tissue and liver. This reduction may impair taurine’s efficacy in improving metabolic health. Moreover, obesity-related metabolic disorders may create a physiological environment that is less responsive and/or slower to adapt to taurine intervention [50]. This could explain why overweight individuals exhibit more significant reductions in weight and BMI when given the same dose of taurine compared to obese individuals. Additionally, differences in genetics and epigenetics between overweight and obese individuals may impact the efficacy of taurine supplementation [51,52,53]. The current research is limited in scope, potentially restricting the generalizability of these findings. To optimize the efficacy of taurine in enhancing metabolic health in overweight and obese individuals, future studies should investigate variations in obesity stages and metabolic condition, and the relative dosage of taurine in relation to body weight should be considered.

Through a meta-analysis of the included RCTs, we found that long-term taurine supplementation significantly promotes glycemic control in obese individuals. Specifically, our findings showed that extended taurine supplementation significantly decreases FPG and HbA1c levels in adults with obesity. The reduction in HbA1c, the gold standard indicator of long-term glycemic control, suggests that taurine contributes to blood glucose stabilization in obese individuals. The decrease in FPG further confirmed the efficacy of taurine in enhancing immediate glycemic control. However, these results contrast with those of a recent meta-analysis that evaluated the impact of taurine on lipid profiles and blood pressure in obese individuals [23]. Guan L et al. [23] reported that taurine supplementation reduced TG and TC but had no significant effect on FPG. This discrepancy may be due to differences in the populations examined. Although focusing on obesity, their study included individuals with hepatitis, cystic fibrosis, and chronic alcoholism who were not overweight or obese and whose blood glucose levels were normal. Such heterogeneity may have affected the consistency of results across studies. Furthermore, the meta-analysis found no significant improvement in glycemic control in overweight individuals after taurine supplementation. This may be related to physiological and clinical distinctions between overweight and obese individuals. Compared with the overweight people, obese individuals typically exhibit more severe metabolic disorders, such as higher FPG levels. This difference might have contributed to the greater improvement in glycemic control observed in obese participants receiving taurine supplementation [19,21,42,43].

Moreover, the dosage of taurine utilized in the available included studies was further analyzed. In human clinical trials, the recommended intake of taurine is 3 g/day [54]. In the included RCTs, taurine was administered at doses ranging from 1 g to 3 g per day. We observed a substantial improvement in HbA1c and FPG levels only when taurine consumption reached 3 g per day; intake below 3 g had no meaningful effect. Consistent with our findings, a meta-analysis evaluating taurine’s impact on the risk of metabolic syndrome demonstrated a dose-dependent relationship between taurine supplementation and improved FPG—that is, the improvement in FPG decreased as the taurine supplementation dose decreased [46]. This suggests the existence of a dose threshold for the effect of taurine in promoting glycemic control, with a supplementation of 3 g of taurine per day potentially necessary for effective glycemic regulation. Nonetheless, the taurine supplementation used in the present included studies, particularly when administered as a single dose, and the lack of tracking of the dietary taurine intake, may have influenced the results. In addition, higher doses (6 g/day) of taurine have been used in some clinical trials to treat patients with chronic alcohol-related disorders or hypertension. However, there are no relevant studies on overweight/obese individuals [55,56]. In conclusion, beyond considering long-term safety and potential adverse effects (such as gastrointestinal discomfort), future research on taurine’s application in blood glucose management for overweight and obese individuals should focus on dose optimization. This involves establishing taurine supplementation levels relative to body weight, tailoring the dosage to individual body mass to maximize effectiveness [57].

The current meta-analysis indicates that taurine significantly reduces insulin resistance in obesity people but has little effect in those who are overweight. This disparity may be related to the more severe insulin resistance present in obesity. Insulin resistance is a central component of the obesity-induced metabolic abnormalities, such as hyperglycemia and hyperlipidemia [58]. In contrast, overweight individuals typically exhibit less insulin resistance, although they may still have some degree of metabolic dysfunction. The results also confirmed (as shown in Figure S4) that overweight adults had lower baseline FPG and less insulin resistance than obese adults. The reductions in HbA1c and FPG observed in obese individuals following taurine supplementation further supports its positive effect on insulin sensitivity in obesity. Interestingly, our findings are inconsistent with those of a recent study [46]. Tzang CC et al. [46] found that taurine significantly improved the HOMA-IR (WMD: −0.693, [95% CI: −1.133, −0.252], *p* = 0.002) in patients with metabolic syndrome, but had no significant effect on HbA1c (WMD: −0.341%, [95% CI: −0.709, −0.28], *p* = 0.07). Their study included patients with various conditions, including heart failure, T2DM, and coronary heart disease. Variations in the disease stages and treatment history may influence taurine’s effectiveness [46]. In contrast, another meta-analysis investigating taurine supplementation on metabolic indicators in diabetes showed that taurine intervention significantly improved HOMA-IR (SMD −0.64 [95% CI: −1.22, −0.06], *p* = 0.03) and fasting insulin (SMD −0.48 [95% CI: −0.99, 0.03], *p* = 0.06) in diabetic patients [57]. This suggests complexity and variability in the effects of taurine on individuals in different metabolic states. Obesity and diabetes frequently coexist as complications [59], and both are associated with chronic hyperglycemia and insulin resistance. Taurine may alleviate insulin resistance by protecting pancreatic β-cells, reducing the inflammatory response, and regulating lipid metabolism [60]. Therefore, taurine may be particularly suitable as an adjunctive treatment for patients with severe metabolic dysfunction, such as those with pronounced insulin resistance associated with obesity and diabetes. These findings align with our results regarding the effects of taurine on obese individuals. In addition, metabolic heterogeneity may underlie why some overweight individuals have not yet developed insulin resistance or are not responsive to taurine intervention [61,62]. In summary, taurine has a minimal influence on insulin sensitization in individuals with minor metabolic disorders (such as being overweight), but exerts a more pronounced effect in those with severe insulin resistance (such as obesity or diabetes).

To our knowledge, this is the first meta-analysis to thoroughly evaluate the effects of long-term taurine supplementation on markers of glycolipid metabolism in individuals who are overweight or obesity. However, this study still has some limitations. For example, the number of eligible RCTS is small due to the lack of large clinical trials (previous studies have focused more on the ergogenic effects of taurine intake on exercise performance). Another limitation of this meta-analysis is that the study population included individuals who were both obese and had T2DM. Metabolic abnormalities associated with T2DM, such as severe insulin resistance and chronic hyperglycemia, could lead to distinct responses to taurine supplementation compared to individuals without diabetes [63,64]. However, the limited availability of studies prevented further subgroup analysis to evaluate the specific effects of taurine in diabetic versus non-diabetic populations. Future research should address this gap by conducting targeted investigations to better understand the role of T2DM in improving glycemic and lipid metabolism with taurine supplementation. In addition, the accuracy of our results could have been affected by confounding factors such as dietary habits, physical activity, and ethnic variations, which were not controlled for in this systematic review and meta-analysis.

## 5. Conclusions

Long-term taurine supplementation provides significant benefits for lipid metabolism in overweight and obese adults. Notably, taurine exhibited greater positive effects on insulin sensitivity and glycemic control in obese people. Furthermore, taurine’s effectiveness may vary depending on the degree of obesity, and a daily intake of 3 g of taurine might significantly improve glycemic management.

## Data Availability

This study did not generate or analyze new data.

## Supplementary Materials

The following supporting information can be downloaded at: https://www.mdpi.com/article/10.3390/nu17010055/s1, Figure S1: Forest plot of standardized mean difference for taurine intake on weight; Figure S2: Forest plot of standardized mean difference for taurine intake on LDL-C; Figure S3: Forest plot of standardized mean difference for taurine intake on HDL-C; Figure S4: Forest plot of standardized mean difference for taurine intake on FBS; Figure S5: Forest plot of standardized mean difference for taurine intake on FBS; Figure S6: Forest plot of standardized mean difference for taurine intake on fasting insulin; Figure S7: Risk of bias summary: review authors’ judgements about each risk of bias item for each included study; Figure S8: Risk of bias graph: review authors’ judgements about each risk of bias item.

## Abbreviations

WHO: World Health Organization; BMI, body mass index; T2DM, type 2 diabetes mellitus; PRISMA, preferred reporting items for systematic reviews and meta-analyses; PROSPERO, International Prospective Register of Systematic Reviews; PICOS, population, intervention, comparator, outcome, study design; RCT, randomized controlled trial; WMD, weighted mean difference; CI, confidence interval; TG, triglycerides; TC, total cholesterol; LDL-C, low-density lipoprotein cholesterol; HDL-C, high-density lipoprotein cholesterol; HbA1c, glycated hemoglobin; FPG, fasting plasma glucose; HOMA-IR, homeostatic model assessment for insulin resistance; PPARα, peroxisome proliferator-activated receptor α; PPARγ, peroxisome proliferator-activated receptor γ; PGC-1α, peroxisome proliferator-activated receptor gamma coactivator-1α; QS, Qin Sun; JPW, Jieping Wang; HYW, Huanyu Wang; HHY, Hanhan Yu; KW, Kang Wan; FYM, Fuyi Ma; RW, Ru Wang.
