# Supplementary material for: Effect of Long-Term Taurine Supplementation on the Lipid and Glycaemic Profile in Adults with Overweight or Obesity: A Systematic Review and Meta-Analysis
Source: Nutrients. 2024 Dec 27;17(1):55. doi: 10.3390/nu17010055 (PMC11722866; doi:10.3390/nu17010055)

Supplementary Figure S1. Forest plot of standardized mean difference for taurine intake on weight. 95%CI = 95% confidence interval; *df* = degree of freedom; I^2 = inconsistency between studies; WMD = standardized mean difference.

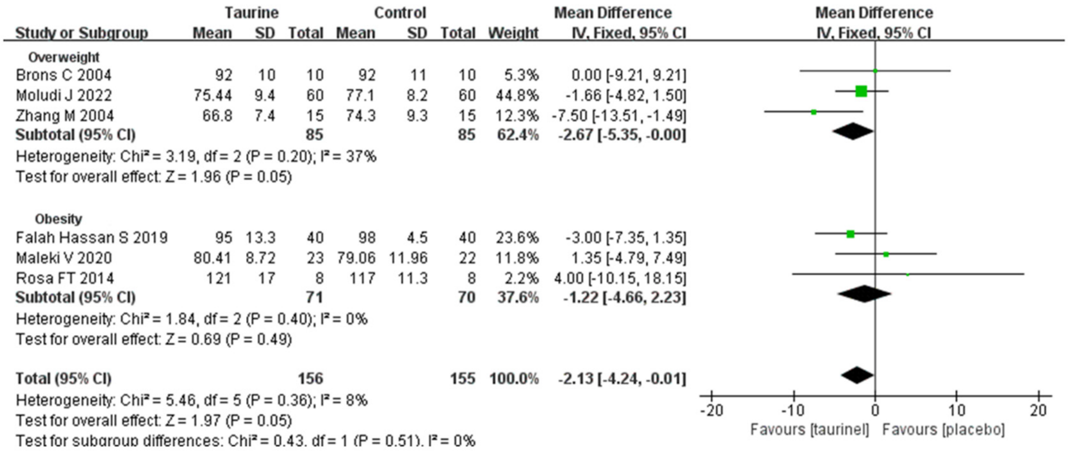

Supplementary Figure S2. Forest plot of standardized mean difference for taurine intake on LDL-C. 95%CI = 95% confidence interval; *df* = degree of freedom; I^2 = inconsistency between studies; LDL-C=low-density lipoprotein; WMD = standardized mean difference.

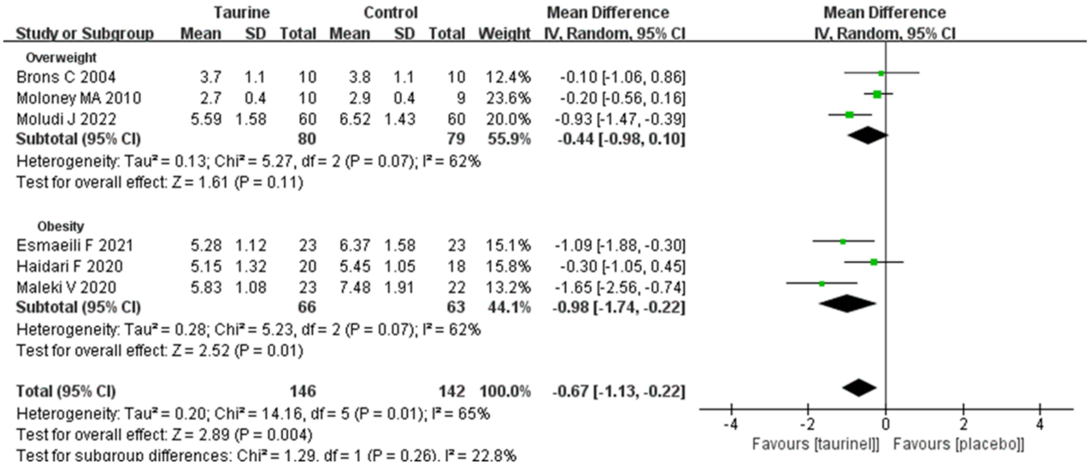

Supplementary Figure S3. Forest plot of standardized mean difference for taurine intake on HDL-C. 95%CI = 95% confidence interval; *df* = degree of freedom; I^2 = inconsistency between studies; HDL-C=high-density lipoprotein; WMD = standardized mean difference.

Study or Subgroup	Taurine Mean	Taurine SD	Taurine Total	Control Mean	Control SD	Control Total	Weight	Mean Difference IV, Random, 95% CI
**Overweight**								
Brons C 2004	1	0.3	10	1	0.2	10	8.8%	0.00 [-0.22, 0.22]
Moloney MA 2010	1.4	0.1	10	1.2	0.1	9	18.1%	0.20 [0.11, 0.29]
Moludi J 2022	2.55	0.4	60	2.43	0.49	60	12.6%	0.12 [-0.04, 0.28]
Zhang M 2004	1.3	0.13	15	1.17	0.17	15	16.6%	0.13 [0.02, 0.24]
**Subtotal (95% CI)**			**95**			**94**	**56.1%**	**0.15 [0.09, 0.21]**
Heterogeneity: Tau² = 0.00; Chi² = 3.18, df = 3 (P = 0.36); I² = 6%								
Test for overall effect: Z = 4.58 (P < 0.00001)								
**Obesity**								
Esmaeili F 2021	2.24	0.1	23	2.23	0.07	23	21.1%	0.01 [-0.04, 0.06]
Haidari F 2020	2.83	0.46	20	2.53	0.45	18	6.2%	0.30 [0.01, 0.59]
Maleki V 2020	2.28	0.17	15	2.33	0.13	15	16.6%	-0.05 [-0.16, 0.06]
**Subtotal (95% CI)**			**58**			**56**	**43.9%**	**0.02 [-0.09, 0.13]**
Heterogeneity: Tau² = 0.00; Chi² = 5.01, df = 2 (P = 0.08); I² = 60%								
Test for overall effect: Z = 0.36 (P = 0.72)								
**Total (95% CI)**			**153**			**150**	**100.0%**	**0.09 [0.00, 0.17]**
Heterogeneity: Tau² = 0.01; Chi² = 22.14, df = 6 (P = 0.001); I² = 73%								
Test for overall effect: Z = 1.98 (P = 0.05)								
Test for subgroup differences: Chi² = 4.25, df = 1 (P = 0.04), I² = 76.5%								

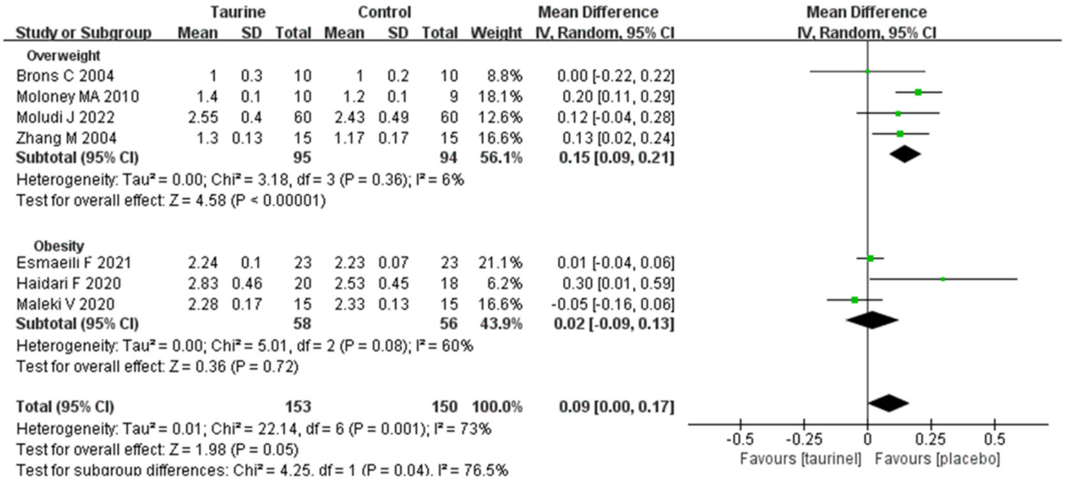

Supplementary Figure S4. Forest plot of standardized mean difference for taurine intake on FBS. 95%CI = 95% confidence interval; *df* = degree of freedom; I^2 = inconsistency between studies; FBS = fasting blood sugar; WMD = standardized mean difference.

Study or Subgroup	Taurine Mean	Taurine SD	Taurine Total	Control Mean	Control SD	Control Total	Weight	Mean Difference IV, Random, 95% CI
**Overweight**								
Brons C 2004	84.6	3.6	10	88.2	3.6	10	19.3%	-3.60 [-6.76, -0.44]
Moloney MA 2010	198	68.4	9	162	61.2	10	0.6%	36.00 [-22.62, 94.62]
Moludi J 2022	123.81	54.22	60	142.43	45.07	60	4.9%	-18.62 [-36.46, -0.78]
Zhang M 2004	77.4	11.52	15	79.2	12.24	15	12.1%	-1.80 [-10.31, 6.71]
**Subtotal (95% CI)**			**94**			**95**	**36.8%**	**-4.16 [-10.31, 1.99]**
Heterogeneity: Tau² = 14.14; Chi² = 4.64, df = 3 (P = 0.20); I² = 35%								
Test for overall effect: Z = 1.32 (P = 0.19)								
**Obesity**								
Esmaeili F 2021	163.83	22.55	23	181.71	20.36	23	8.1%	-17.88 [-30.30, -5.46]
Falah Hassan S 2019	121.5	9.8	40	122.6	12.2	40	17.1%	-1.10 [-5.95, 3.75]
Haidari F 2020	77.25	9.08	20	74.77	9.85	18	15.4%	2.48 [-3.56, 8.52]
Maleki V 2020	155.99	17.62	23	176.06	21.83	22	8.8%	-20.07 [-31.69, -8.45]
Rosa FT 2014	85	5.65	8	92	8.49	8	13.9%	-7.00 [-14.07, 0.07]
**Subtotal (95% CI)**			**114**			**111**	**63.2%**	**-7.16 [-14.45, 0.14]**
Heterogeneity: Tau² = 51.06; Chi² = 18.59, df = 4 (P = 0.0009); I² = 78%								
Test for overall effect: Z = 1.92 (P = 0.05)								
**Total (95% CI)**			**208**			**206**	**100.0%**	**-5.60 [-10.09, -1.12]**
Heterogeneity: Tau² = 24.59; Chi² = 23.23, df = 8 (P = 0.003); I² = 66%								
Test for overall effect: Z = 2.45 (P = 0.01)								
Test for subgroup differences: Chi² = 0.38, df = 1 (P = 0.54), I² = 0%								

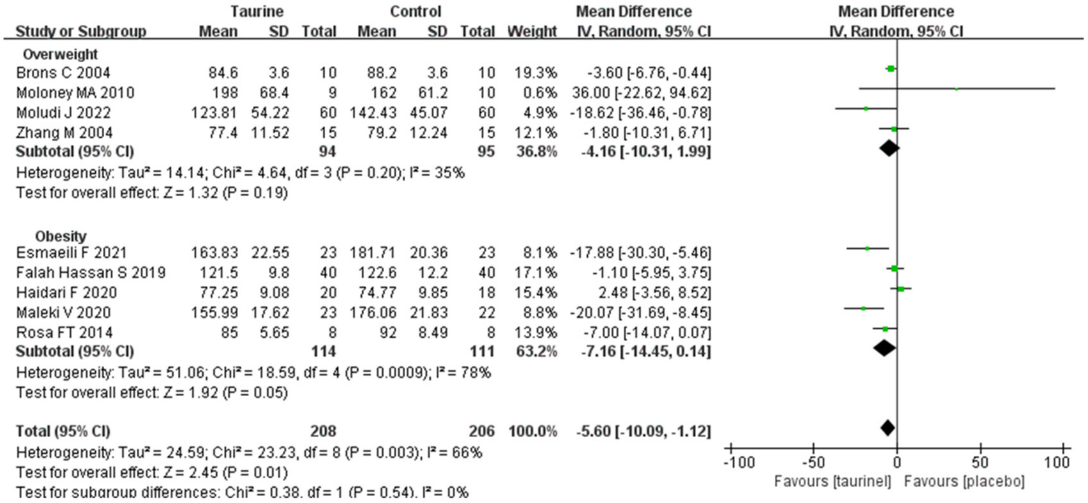

Supplementary Figure S5. Forest plot of standardized mean difference for taurine intake on FBS. 95%CI = 95% confidence interval; *df* = degree of freedom; I^2 = inconsistency between studies; FBS = fasting blood sugar; WMD = standardized mean difference.

Study or Subgroup	Taurine Mean	Taurine SD	Taurine Total	Control Mean	Control SD	Control Total	Weight	Mean Difference IV, Random, 95% CI
**3g taurine**								
Brons C 2004	84.6	3.6	10	88.2	3.6	10	19.3%	-3.60 [-6.76, -0.44]
Esmaeili F 2021	163.83	22.55	23	181.71	20.36	23	8.1%	-17.88 [-30.30, -5.46]
Haidari F 2020	77.25	9.08	20	74.77	9.85	18	15.4%	2.48 [-3.56, 8.52]
Maleki V 2020	155.99	17.62	23	176.06	21.83	22	8.8%	-20.07 [-31.69, -8.45]
Moludi J 2022	123.81	54.22	60	142.43	45.07	60	4.9%	-18.62 [-36.46, -0.78]
Rosa FT 2014	85	5.65	8	92	8.49	8	13.9%	-7.00 [-14.07, 0.07]
Zhang M 2004	77.4	11.52	15	79.2	12.24	15	12.1%	-1.80 [-10.31, 6.71]
**Subtotal (95% CI)**			**159**			**156**	**82.4%**	**-7.14 [-12.53, -1.74]**
Heterogeneity: Tau² = 32.06; Chi² = 20.05, df = 6 (P = 0.003); I² = 70%								
Test for overall effect: Z = 2.59 (P = 0.009)								
**<3g taurine**								
Falah Hassan S 2019	121.5	9.8	40	122.6	12.2	40	17.1%	-1.10 [-5.95, 3.75]
Moloney MA 2010	198	68.4	9	162	61.2	10	0.6%	36.00 [-22.62, 94.62]
**Subtotal (95% CI)**			**49**			**50**	**17.6%**	**5.48 [-22.29, 33.25]**
Heterogeneity: Tau² = 237.95; Chi² = 1.53, df = 1 (P = 0.22); I² = 35%								
Test for overall effect: Z = 0.39 (P = 0.70)								
**Total (95% CI)**			**208**			**206**	**100.0%**	**-5.60 [-10.09, -1.12]**
Heterogeneity: Tau² = 24.59; Chi² = 23.23, df = 8 (P = 0.003); I² = 66%								
Test for overall effect: Z = 2.45 (P = 0.01)								
Test for subgroup differences: Chi² = 0.76, df = 1 (P = 0.38), I² = 0%								

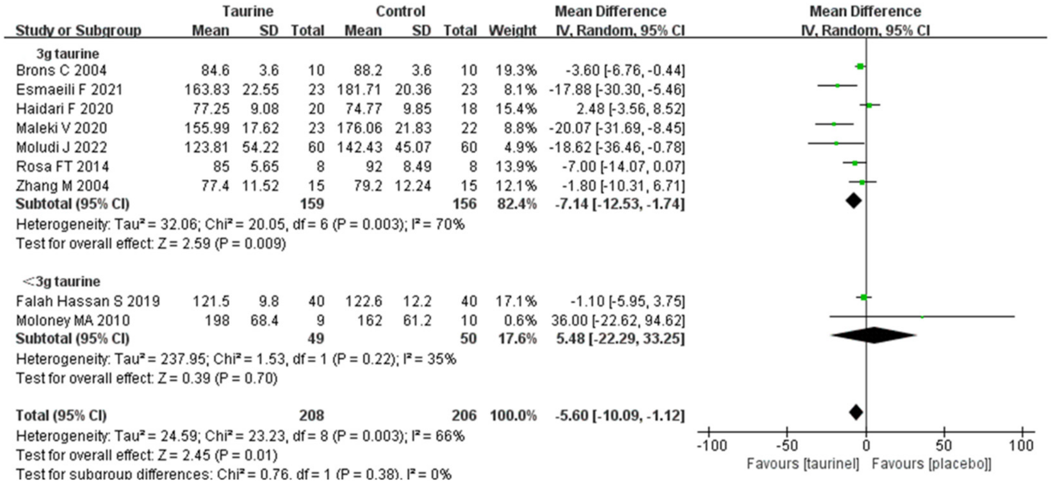

Supplementary Figure S6. Forest plot of standardized mean difference for taurine intake on fasting insulin. 95%CI = 95% confidence interval; *df* = degree of freedom; I^2 = inconsistency between studies; WMD = standardized mean difference.

Study or Subgroup	Taurine Mean	Taurine SD	Taurine Total	Control Mean	Control SD	Control Total	Weight	Mean Difference IV, Random, 95% CI
Esmaeili F 2021	10.01	3.48	23	11.48	3.46	23	25.9%	-1.47 [-3.48, 0.54]
Haidari F 2020	10.02	6.17	20	10.5	5.36	18	8.5%	-0.48 [-4.15, 3.19]
Maleki V 2020	10.23	3.27	23	12.17	3.11	22	29.4%	-1.94 [-3.80, -0.08]
Moludi J 2022	10.36	4.31	60	13.35	5.05	60	35.2%	-2.99 [-4.67, -1.31]
Rosa FT 2014	15	5.65	8	25	14.14	8	1.1%	-10.00 [-20.55, 0.55]
**Total (95% CI)**			**134**			**131**	**100.0%**	**-2.15 [-3.24, -1.06]**
Heterogeneity: Tau² = 0.14; Chi² = 4.37, df = 4 (P = 0.36); I² = 9%								
Test for overall effect: Z = 3.87 (P = 0.0001)								

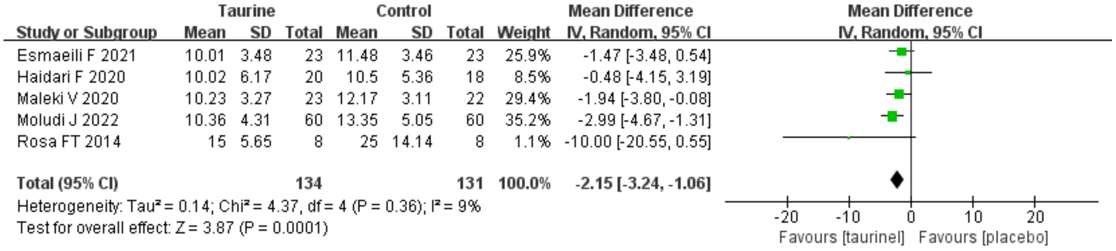

Supplementary Figure S7. Risk of bias summary: review authors' judgements about each risk of bias item for each included study.

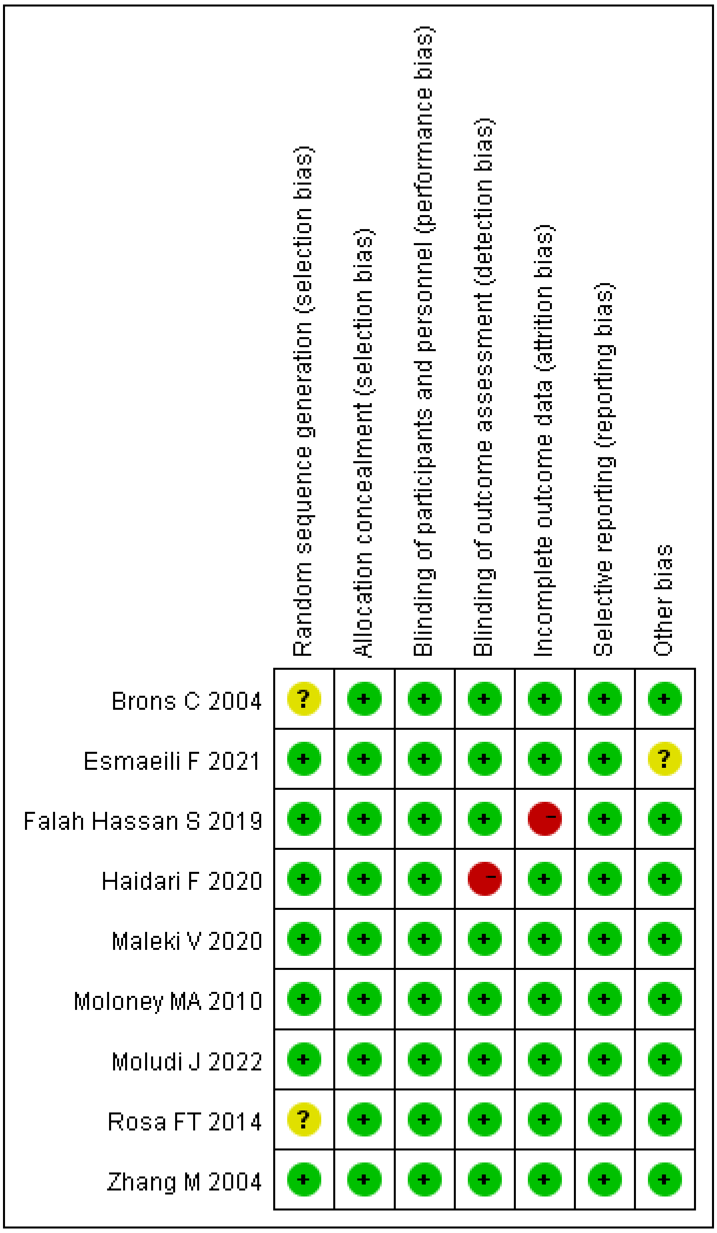

Supplementary Figure S8. Risk of bias graph: review authors' judgements about each risk of bias item.

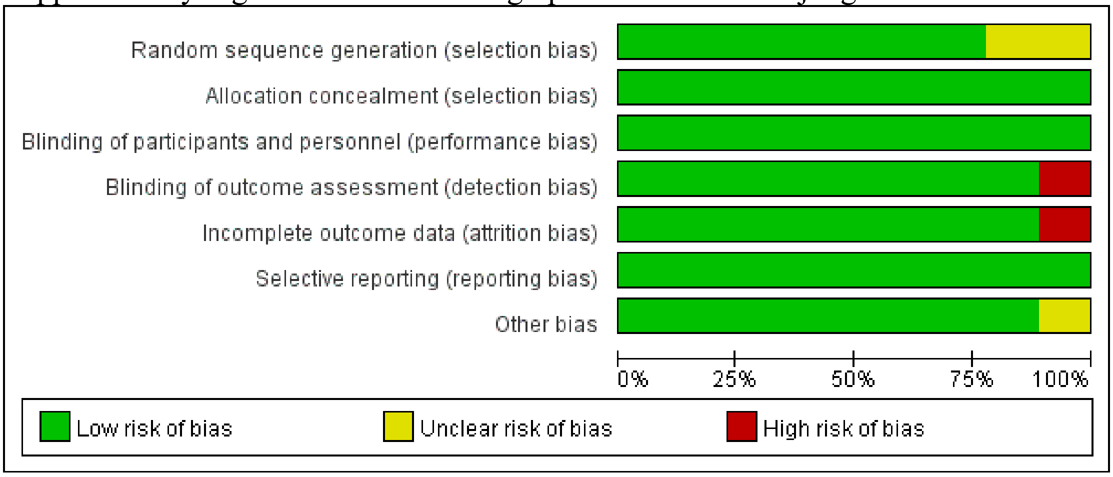

Supplement: Supplementary file 1 [file nutrients-17-00055-s001.zip › nutrients-3373004-supplementary.pdf]
